# Genome wide data recover hierarchical genetic structure and help define conservation units for the threatened Asian Houbara

**DOI:** 10.1038/s41598-025-33691-3

**Published:** 2026-01-28

**Authors:** Thierry Bernard Hoareau, Keiler Arthur Collier, Matthew J. Miller, Yves Hingrat, Eric Le Nuz, Nyambayar Batbayar, Ohad Hatzofe, Asaf Mayrose, Loïc Lesobre

**Affiliations:** 1 Reneco International Wildlife Consultants LTD, Abu Dhabi, United Arab Emirates; 2 Wildlife Science and Conservation Center of Mongolia, Ulaanbaatar, Mongolia; 3 Division of Science & Conservation, Israel Nature & Parks Authority, Jerusalem, Israel; 4 Department of Evolutionary and Environmental Biology and Institute of Evolution, University of Haifa, Haifa, Israel

**Keywords:** Adaptive evolutionary conservation, Conservation strategy, DAPC, Partially migratory species, Runs of homozygosity, Whole genome sequencing, Population genetics, Conservation biology

## Abstract

The Asian Houbara Bustard (*Chlamydotis macqueenii*), a partially migratory bird from the western and Central Asian steppes, is listed as vulnerable on the IUCN Red List. This study reassesses the species’ genetic structure using modern genomics to identify evolutionary significant units (ESUs). Following the generation of a de novo reference assembly and resequencing data (114 birds, 10 locations), we integrated genetic results, migratory behaviour, and geography to identify eight hierarchically structured ESUs: four near range edges (Yemen, Mongolia, Eastern Kazakhstan, Israel) and four within the central range (Central-Eastern, Central-Western, North Iran, South Iran). Low genetic diversity and recent inbreeding make ESUs on the range periphery (Israel, Mongolia, Yemen) the most genetically threatened, consistent with the central-marginal hypothesis. ESUs do not cluster according to their migrant/non-migrant status. Geographic distance significantly shaped genetic structure, with longitudinal separation (isolation-by-distance along an east–west axis) emerging as the strongest predictor of differentiation, particularly among high-latitude migrant populations. Our findings underscore the importance of integrating genomic, geographic and behavioural criteria to define intraspecific units that effectively address the conservation needs of widespread species with complex evolutionary dynamics.

**Supplementary Information:**

The online version contains supplementary material available at 10.1038/s41598-025-33691-3.

## Introduction

In the face of rapid environmental changes, understanding the genetic diversity distribution in threatened species is essential for effective conservation strategies, ensuring long-term population viability, minimizing extinction risks, and enhancing adaptability^1^. Identifying distinct conservation units, such as Evolutionary Significant Units (ESUs) introduced by^2^, is crucial for targeting conservation efforts and safeguarding genetic integrity. The ESU concept has evolved to include reproductive isolation, ecological distinctiveness^3^, and both neutral and adaptive genetic variation^4–6^. Fraser and Bernatchez^6^ proposed the Adaptive Evolutionary Conservation (AEC) framework to unify ESU identification, using both genetic and ecological criteria (e.g., physical isolation or adaptive divergence) to delineate species subdivisions. Today, new initiatives propose integrating these flexible identifications of ESUs into IUCN standards to protect overlooked genetic diversity critical for species survival, especially for data-limited species^7^.

Population genetics offers a framework to describe units below the species level, especially in cases where the species exhibits hierarchical structuring^8,9^. Genetic diversity often varies between core and peripheral ranges, leading to different conservation needs across the species’ distribution^10^. In this context, peripheral populations are critical due to their increased vulnerability to threats^11–13^, unique genetic diversity and insights into species responses to environmental changes^10,14,15^ and helps better understand the dynamic interplay between genetics and environments^10^. These populations, isolated by geographic distance or natural barriers, typically exhibit restricted gene flow and increased genetic differentiation, promoting adaptation to new environments^16^. When anthropogenic factors like habitat degradation and climate change are superimposed to these demographic conditions, it increases the extinction risk of these locally adapted reservoirs^17^. Therefore, it is essential to conduct genetic surveys across the range of widespread species, especially in peripheral regions to understand processes that contribute to the species’ overall genetic structure.

**Fig. 1:**
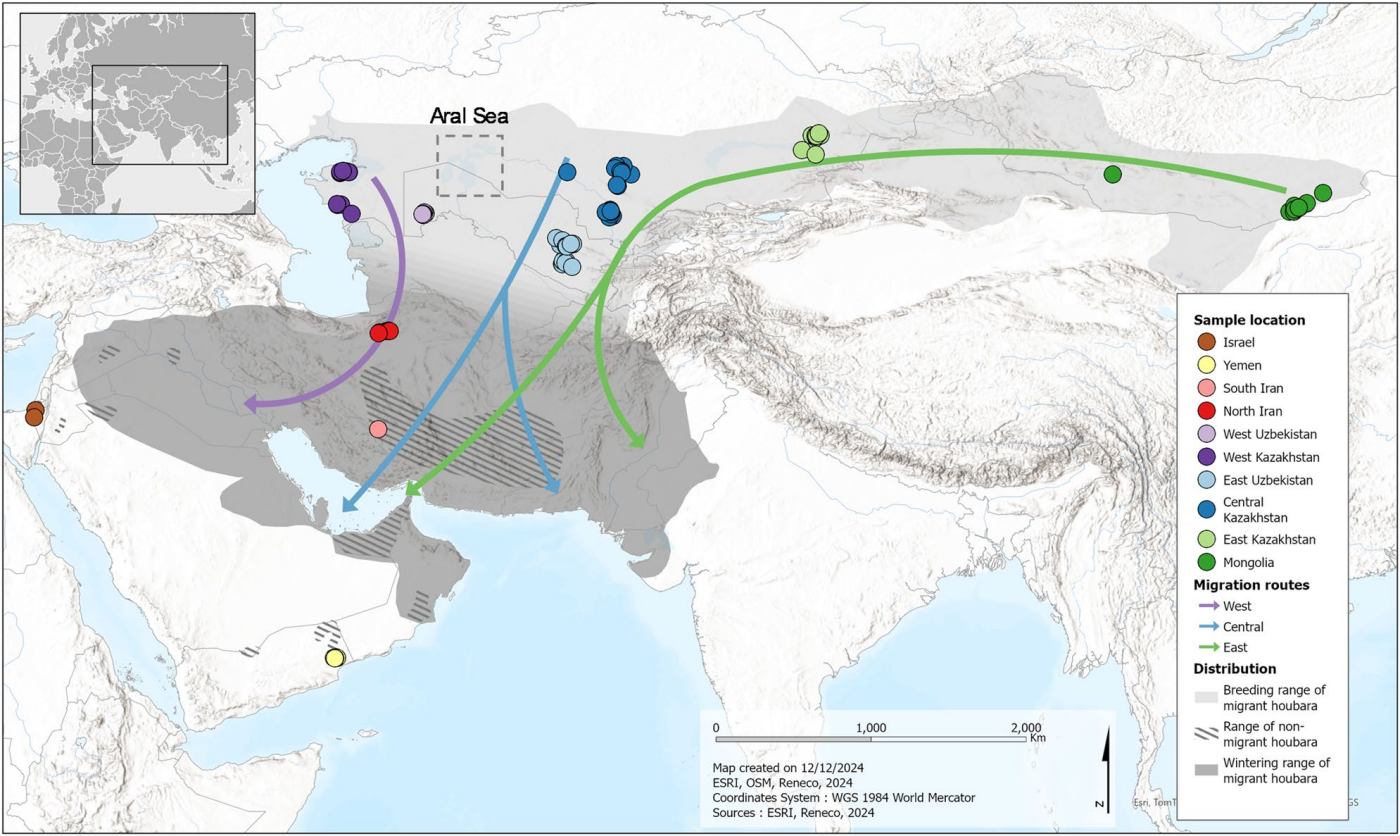
Distribution range of migratory (uniform grey areas) and non-migratory (dark grey cross hatched areas) Asian Houbara (*C. macqueenii*), including the sampling locations and the three main recognised migration routes depicted by the coloured arrows.

The Asian Houbara Bustard (*Chlamydotis macqueenii*) plays a significant role in traditional falconry, where it is highly prized as prey for trained falcons, driving unsustainable hunting pressure. This has contributed to its significant decline since the 1970s, leading to its listing as vulnerable on the IUCN Red List^18^ and its inclusion in CITES Appendix I and CMS Appendix II. Its range extends from the Sinai Peninsula across the Middle East and Central Asia to Mongolia (Fig. 1), covering diverse habitats from semi-arid to arid steppe lands, and from low to high-altitude regions^19^. Across this extensive range, ecological and behavioural studies have shown important variations in life history traits between populations, reflecting potential local adaptations and divergences.

Among the traits of *C. macqueenii*, migratory behaviour stands out as a striking example of potential adaptive divergence^19^. This behaviour varies significantly among populations, with non-migratory birds breeding at lower latitudes in the southern and western range, while the migratory individuals are found in central, northern, and eastern range (Fig. 1^19^). According to this study^19^, resident populations may persist in fragmented regions, such as Baluchistan, southern Iran, Oman, Yemen, and Sinai, where natural barriers (deserts, mountains, seas) likely limit genetic exchange among non-migratory groups. A divide in migration pathways and breeding ranges exists around the Aral Sea separating birds following the western and central routes in both their breeding and wintering areas (Fig. 1)^19,20^. These studies also identified a third eastern migration route, where birds breed in the far east and their wintering range overlaps with birds from the central migration route. Furthermore, migration timing, direction, and distances also vary between migration routes^21,22^. These patterns, along with a philopatric behaviour and the fact that juveniles migrate alone before adults^19,22,23^, suggest heritable migration behaviours with a strong genetic basis in *C. macqueenii*. Taken together, geography (distance and barriers) and behaviour indicate multiple sources of gene flow restriction across the range, suggesting the existence of a complex genetic structure.

Since *C. macqueenii* was recognised as a distinct species^24–26^, genetic studies using mtDNA and microsatellites have revealed low genetic differences among locations but no genetic clustering^27,28^. The structuring was primarily attributed to differences between migrant and non-migrant individuals as well as variations in longitude among migrants. Moreover, genetic differences were observed for individuals from Yemen and Western Kazakhstan^28^, as well as from Sinai^27^, indicating locations that are genetically unique or isolated compared to the rest of the distribution. While these seminal studies have been important for understanding the genetic structure of *C. macqueenii*, inconsistencies of results between studies due to marker resolution and sampling limitations hinder the identification of conservation units. This underscores the need for advanced genomic data to refine conservation strategies.

To ensure the persistence of *C. macqueenii*, multiple ex-situ conservation programmes have been established, some leveraging insights from past genetic and migration studies. For example, the International Fund for Houbara Conservation (IFHC; https://houbarafund.gov.ae/) manages the species based on both geographic origin and migratory behaviours derived from earlier studies^19,27,28^. While these previous studies remain relevant, the lack of clearly defined conservation units highlights the need to update and refine our understanding of population structure and genetic status using the latest genomic tools. Using whole-genome resequencing (WGS) data from a sample of individuals covering the species’ range, including peripheral and central locations, migrants and non-migrants, as well as individuals of all known migratory routes, this study aims to comprehensively define conservation units of *C. macqueenii* (i.e. ESUs) across its range. After producing a reference genome assembly for the species, we analysed WGS data to identify distinct genetic clusters across the range. Our underlying hypotheses derived from previous studies^19,27,28^ propose that differences between migrants and non-migrants, flyway divergence, and longitudinal variations among migrants are creating a hierarchical genetic structure. Combining these insights to the AEC framework^6^, we then propose conservation units before assessing their conservation status based on geographic positions, genetic distinctness, habitat specificities, and migratory behaviours. This comprehensive approach aims to provide a robust framework to inform conservation strategies for *C. macqueenii*, ensuring their survival and sustainability across their distribution range.

## Results

### Reference genome assembly

From 11 sequencing libraries, we obtained ~ 97.3 Gbp of trimmed data, resulting in 6.11 million reads and an average coverage of 34x. The assembly included 868 contigs with a contig N50 size of 21.10 Mb, an L50 of 16, and a total genome size of 1.16 Gbp, consistent with the 1.30–1.08 Gbp range of prior short-read assemblies for the species. A total of 97% of the genome assembly consists of ungapped contigs over 1 Mbp. We recovered 96.6% (8,058/8,338) complete single-copy avian BUSCO genes, with 0.9% (79) as fragments and 2.5% (201) missing. Taken together, these statistics suggest a relatively complete assembly with sufficient contiguity to assess long (> 1Mbp) ROH, particularly in complex regions of the genome.

### Resequencing, mapping and variant calling

We analysed 114 individuals from 10 locations, averaging 11 individuals per location (range: 5–21; Table 1). After quality filtering, sequencing yielded an average of 9.7 million short reads per sample (range: 3.1–53.9 million), with mapping rates over 97% to the new *C. macqueenii* reference genome, and average read depth per individual of 24.5× (range: 16×–39.5×). After the first round of filtering, we identified 4,476,589 SNPs (SNPset#1) for diversity and ROH analyses. We also obtained 90,829 SNPs (SNPset#2) after the second round of filtering. SNPset#2 was used for *Fst*, DAPC, and ADMIXTURE analyses.

**Table 1:** Details of sampling locations and status (migrant or non-migrant) of individuals C. *macqueenii* used in the study. Sampling was conducted between 2003 and 2022.

Sampling location	Status	N	Males	Females	Latitude	Longitude
Israel	Non-migrant	5	1	4	30.95	34.60
Yemen	Non-migrant	7	3	4	16.87	52.03
South Iran	Non-migrant	10	7	3	30.07	54.48
North Iran	Migrant	8	5	3	35.76	54.87
West Uzbekistan	Migrant	8	8	0	42.60	57.15
East Uzbekistan	Migrant	17	3	14	40.29	65.35
Central Kazakhstan	Migrant	21	16	5	43.99	68.18
West Kazakhstan	Migrant	13	10	3	44.01	52.50
Eastern Kazakhstan	Migrant	11	3	8	46.93	79.79
Mongolia	Migrant	14	14	0	43.14	107.09
Total		114	70	44		

### Genetic diversity and level of inbreeding

**Fig. 2:**
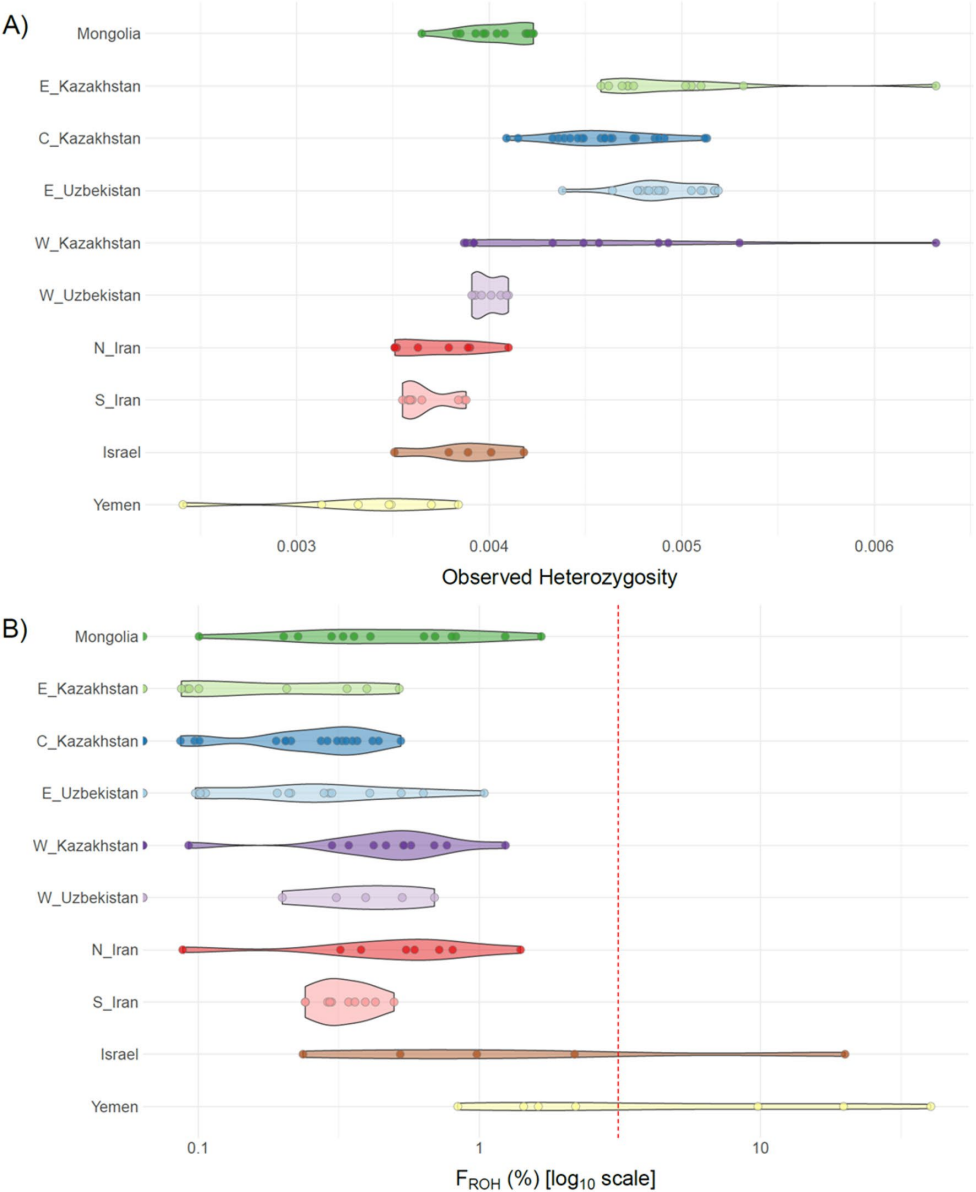
Observed heterozygosity (**A**) and percent of genome found in Runs of Homozygosity (**B**) for each location of C. macqueenii; the red dashed line in B represents the expected percent of genome found in Runs of Homozygosity for the progeny of first cousins in a large population in Hardy–Weinberg equilibrium.

Non-migratory individuals from Yemen had the lowest observed heterozygosity (Fig. 2A; Table S1) while the highest heterozygosity was in East Uzbekistan and West, Central, and East Kazakhstan; with East and West Kazakhstan showing higher variance. Intermediate values were found in Israel, South Iran, North Iran, West Uzbekistan, and Mongolia. Individual inbreeding coefficients, calculated from genome-wide runs of homozygosity (*Froh*), revealed elevated values in Yemeni and Israeli populations compared to central Asian groups (Kazakhstan and Uzbekistan; Fig. 2B). Bootstrap validation confirmed that diversity and inbreeding estimates were robust to uneven sample sizes, with mean heterozygosity and *Froh* values stabilising rapidly regardless of subsampling sizes (Fig. S1). While smaller sample sizes (e.g., Israel) showed wider confidence intervals, no systematic bias was observed in diversity or ROH metrics.

### Delimitation of genetic clusters

**Fig. 3:**
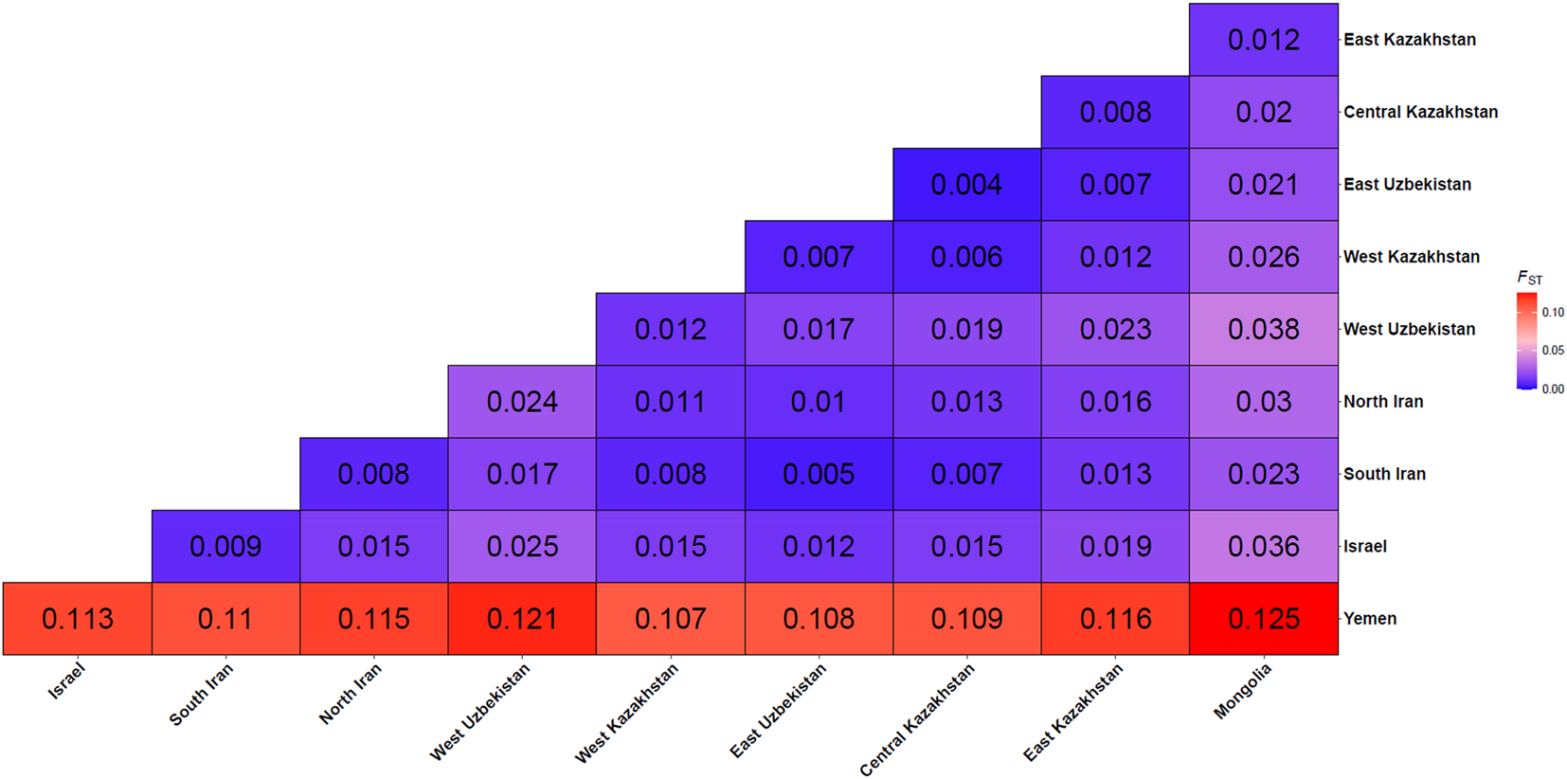
*FST* heatmap obtained for each pair of Asian Houbara (C. *macqueenii*) sampling locations.

**Fig. 4:**
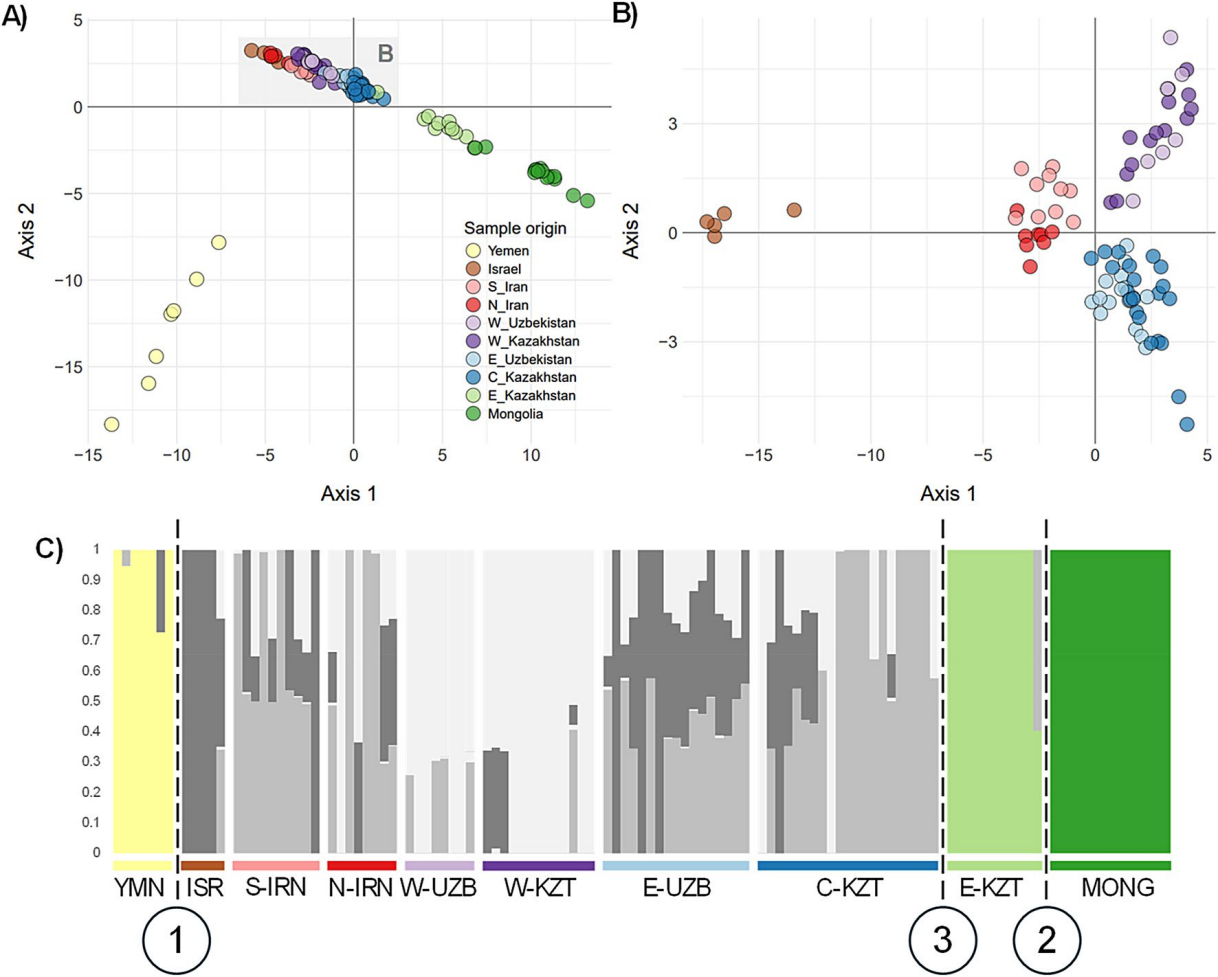
Population structure of Asian Houbara (*C. macqueenii*) depicted through DAPC (**A** and **B**), and Admixture analysis (**C**). (**A**) DAPC approach applied to individuals from all locations; the Greater Western and Central Asian region encompassing Central and West Kazakhstan, East and West Uzbekistan, South and North Iran and Israel is illustrated by the shaded rectangle. (**B**) DAPC focusing on the Greater Western and Central Asian region, primarily situated near the centre of the distribution range. (**C**) Admixture results obtained via our iterative approach. Test 1 reveals Yemen as distinct (where K = 2 is the most likely according to the delta K procedure applied when all locations are included), followed by Mongolia in Test 2 (where K = 2 is the most likely after excluding Yemen from the dataset), and Eastern Kazakhstan in Test 3 (where K = 2 is the most likely after excluding Yemen and Mongolia). Subsequently, we illustrate the remaining individuals for K = 3 to elucidate genetic differences and similarities among locations, but the delta K procedure is unable to further discriminate the number of genetic clusters.

Complimentary analyses using *Fst*, DAPC and Admixture methods, revealed hierarchical population structure, with Yemen, Mongolia, Eastern Kazakhstan as distinct from units found in the Greater Western and Central Asian region (Table 2; Figs. 3 and 4). Initial *Fst* values ranged from 0.003 to 0.115 and were significant, with the highest values in Yemen, followed by Mongolia and Israel (Fig. 3). Subsequent DAPC and Admixture analyses (K = 2) of all populations confirmed the primary division of Yemen. After excluding Yemen, DAPC and Admixture (K = 2) highlighted Mongolia as the next most distinct group. Further exclusion of Mongolia revealed Eastern Kazakhstan’s differentiation through elevated pairwise *Fst* values and distinct DAPC and Admixture clustering (K = 2). DAPC revealed four additional genetic clusters in the Greater Western and Central Asian region (Fig. 4A): (1) Central Kazakhstan/East Uzbekistan, (2) Western Kazakhstan/Western Uzbekistan, (3) North and South Iran, and (4) Israel, with Israel showing the highest *Fst* difference between these locations. Although the ΔK method failed to detect finer-scale population structure among samples from the Greater Western and Central Asian region, Admixture analysis at K = 4 confirmed that individuals from each location exhibited ancestry proportions aligned with the clusters identified by DAPC (Fig. 4; Fig. S2). This stepwise approach uncovered nested genetic patterns, where dominant divisions masked finer-scale structure. When combining these genetic criteria with behavioural traits (Non migrant, and Western, Central and Eastern migration route), we obtained eight ESUs (Table 2). The west–east gradient in migratory locations corresponded to genetic clustering identified by DAPC, with distinct clusters aligning to specific migratory routes (Fig. 4B).

**Table 2:**
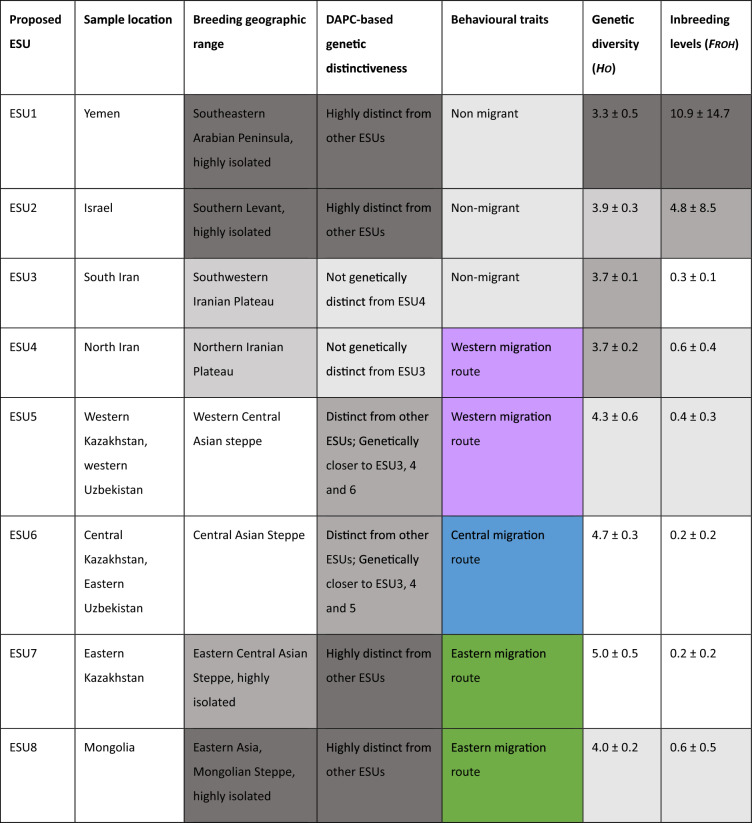
Specificities and genetic status of the proposed ESUs for *C. macqueenii* across its distribution range. The cells are shaded using a gradient to reflect genetic diversity and inbreeding levels: darker cells indicates high genetic diversity and low inbreeding, while lighter cells indicate low genetic diversity and high inbreeding. Colours for behaviour traits correspond to those used in Fig. 1, distinguishing non‑migrant, migrant, and specific migration routes.

**Fig. 5:**
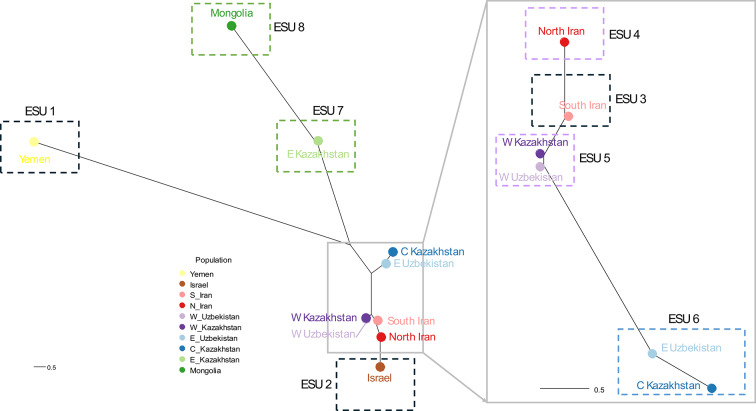
Neighbour-joining dendrogram based on PCA axes 1–2, illustrating genetic relationships among ESUs (dash boxed) and their association with migratory behaviour types (coloured boxes; see Table 2).

The neighbour-joining dendrogram (Fig. 5) reinforced the hierarchical population structure identified by *Fst*, ADMIXTURE, and DAPC analyses. Central Asian ESUs (e.g., Central Kazakhstan/Eastern Uzbekistan, Western Kazakhstan/Western Uzbekistan, and North and South Iran) clustered centrally on the dendrogram, while peripheral ESUs (Yemen, Israel, Mongolia) occupied exterior branches, with Yemen and Mongolia showing the deepest divergences. The topology also reflected migratory ecotypes: ESUs following eastern, central, and western migration routes formed sequential clades across the dendrogram, with the central migratory group positioned intermediately between eastern and western ones. Resident populations (e.g., Yemen, Israel, South Iran) were scattered throughout the dendrogram and did not form a distinct and unique group, confirming that non-migratory traits do not directly predict genetic similarity.

### Geographic correlates of genetic variation in migratory individuals

**Fig. 6:**
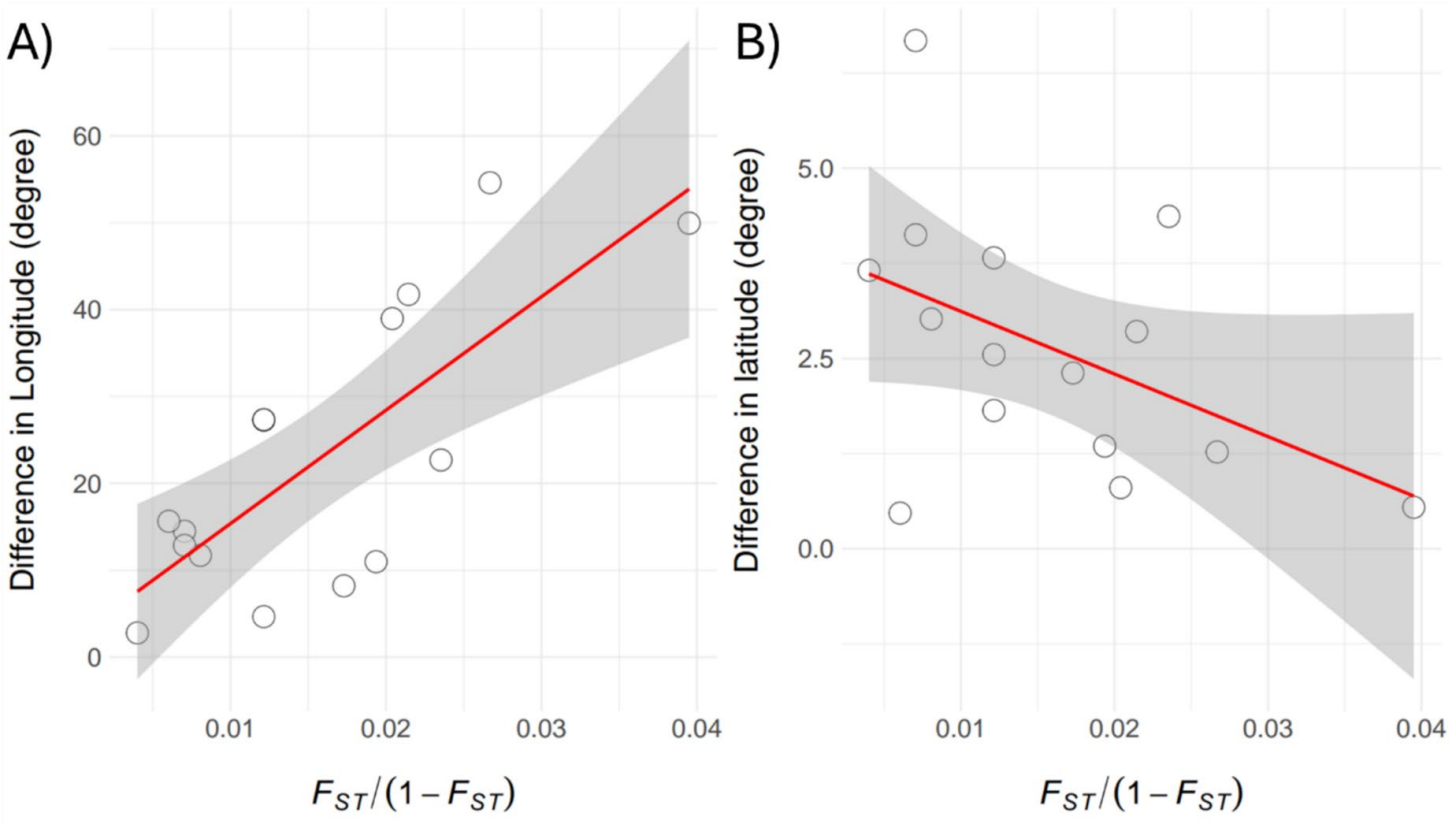
Relationship between genetic distance (*Fst*/(1 − *Fst*)) and longitude (**A**) and latitude differences (**B**) among sample locations of high-latitude migrant individuals (excluding individuals from Northern Iran). The regression analysis for longitude shows a significant positive relationship: ΔL = 2.3031 + 1305.35 × *Fst*/(1 − *Fst*); Spearman’s *ρ* = 0.6475*, P* = 0.0091. In contrast, the analysis for latitude indicates a non-significant negative relationship: Spearman’s* ρ* = − 0.3408, *P* = 0.2138.

Our isolation-by-distance analysis of high-latitude migratory populations (excluding North Iran) revealed a strong positive correlation between longitudinal separation and genetic differentiation (Mantel test: Spearman’s *ρ* = 0.6475, *P* = 0.0091; Fig. 6), indicating increasing genetic divergence along the east–west axis. In contrast, no significant association was detected between genetic distance and latitudinal differences (Spearman’s *ρ* = − 0.3408, *P* = 0.2138). These results suggest longitudinal geographic barriers play a more prominent role than latitudinal ones in shaping population structure.

## Discussion

Identifying conservation units in species with hierarchical genetic structures and varying behavioural traits is challenging, therefore complicating conservation planning. Here, we successfully used whole genome sequencing and the AEC framework^6^ to identify conservation units in the endangered Asian Houbara, *C. macqueenii*, a partially migratory bird. Our hierarchical, multi-criteria approach to ESU delineation aligns with the Adaptive Evolutionary Conservation (AEC) framework^6^, which advocates for integrating genetic and ecological criteria to identify evolutionarily significant units. By combining neutral genetic divergence (*Fst* > 0.05), population structure (ADMIXTURE and DAPC), and ecological distinctiveness (biogeographic isolation and migratory traits), we captured both demographic independence and adaptive potential. This approach is particularly relevant to guide future conservation efforts for data-limited species, as it provides a flexible yet robust framework for identifying ESUs that reflect critical genetic diversity^7^.

### Hierarchical genetic structure in *C. macqueenii* and its potential drivers

We discovered a hierarchical genetic structure in *C. macqueenii*, indicating that geographic and behavioural differences significantly influence gene flow across its range. We first identified substantial genetic differentiation, distinguishing seven genetic clusters (Figs. 4 and 5): Yemen, Mongolia, Eastern Kazakhstan, and a broader group from Greater Western and Central Asia, which was further subdivided into four clusters (Israel, Iran, and locations along the western and central migratory routes). These seven genetically distinct clusters, consistently identified across multiple analytical approaches, represent primary Evolutionarily Significant Units (ESUs) for the species (Table 2, Fig. 5). Though uneven sample sizes may increase variance for smaller locations (e.g., Israel), our bootstrap analyses confirm that the observed genetic diversity patterns reflect biological reality rather than sampling artifacts, reinforcing confidence in the identified ESUs. Integrating genetic divergence, geographic isolation, and migratory behaviour confirmed the seven ESUs.

Birds from North and South Iran are genetically close yet polarised, indicating mild genetic differences (Figs. 1 and 2B; Table 2). However, tracking studies reveal a clear divide in migratory behaviour of these individuals^19^ with northern birds migrating seasonally, while southern birds remain sedentary. The genetic and behavioural decoupling in Iran underscores the need for independent management, thus supporting the designation of two ESUs. This suggests either a recent adaptive divergence, with selection occurring in specific genomic regions not yet reflected in neutral genetic differences, or phenotypic variation across latitudes with differential expression of pre-existing potential, where migratory traits are expressed at higher latitudes but not at lower ones, as seen in other taxa^29^. In the latter scenario, latitude would play a pivotal role in migration, with a potential threshold above which individuals would express the migratory phenotypes. It is crucial to determine whether migration is influenced solely by environmental cues or by a combination of environmental and genetic factors, which might be elucidated through a candidate gene approach^30^.

Defining eight ESUs allows us to identify fine-scale groups that align with the three previously identified migration routes, while also distinguishing multiple ESUs within these routes as well as recognising unique ESUs within non-migrant individuals. Previous studies using traditional markers failed to distinguish genetically homogeneous groups in *C. macqueenii*, showing limited genetic structure across the species’ range. Pitra et al.^27^ found no evidence of historical separations or barriers that would create distinct genetic groups. Riou et al.^28^ identified significant genetic differences in Yemen, Sinai Peninsula, and Western Kazakhstan but attributed them to migrant–non-migrant differences or longitudinal variations among migrants. This contrast highlights potential limitations of past sampling designs and marker resolutions in identifying genetic structures for conservation planning.

Contrary to Riou et al.^28^, our results show that the migrant vs. non-migrant divide is not the best predictor of the overall genetic structure in *C. macqueenii*. While our results show that non-migrants from Yemen are the most genetically distinct across the range as shown previously^28^, non-migrants from Israel and southern Iran are genetically closer to other migrant ESUs than to Yemen (Table 2; Figs. 4 and 5). Similarly, southern Iran is closer to northern Iran migrants than to Israel. Results call for further analyses to investigate the genetic history of migration in these Iranian birds and how environmental factors shape its expression, highlighting the dynamic divergence of migratory behaviours within the species as illustrated in other bird species^31–33^.

The hierarchical structure we found supports the central-marginal hypothesis^10^, where populations become smaller, less diverse, more divergent, and more sensitive to threats towards range edges. The core of the range, within Greater Western and Central Asia, has more diverse and genetically similar clusters, while peripheral clusters like Yemen, Mongolia, and Israel are more divergent and less diverse (Fig. 1, Table 2). Among migrants, the divergence between western, central, and eastern migration routes shows clear genetic differences (Fig. 5), but their hierarchical structure aligns with the stepping-stone model of dispersal^34^, where populations spread gradually from a central point, leading to greater differentiation at the periphery. In line with previous results^28^, our findings show that genetic distance between high-latitude migrant locations is well explained by longitude differences (Fig. 6), supporting this dispersal model and the central-marginal hypothesis^10^. Satellite tracking data reveals strong philopatry in *C. macqueenii* migrants^19^, indicating that geographic factors like longitude and physical barriers can significantly impact population structure, corroborating our genetic evidence.

### Genetic status of non-migratory ESUs in range edges

Our analysis shows that the genetic profile of non-migrant populations of *C. macqueenii* from both Yemen (ESU1) and Israel (ESU2) is potentially detrimental to their long-term survival and adaptability, making them vulnerable to extirpation. Both populations exhibit genetic isolation and significantly reduced genetic diversity, as well as higher inbreeding levels compared to other ESUs, which are known to compromise fitness, reproductive success, and adaptability^1,35^. These genetic features may stem from long-term small population sizes and genetic isolation, resulting from their geographic isolation and human induced threats (habitat degradation and poaching).

Field surveys in Yemen have highlighted the demographic vulnerability of the population, with less than 200 individuals observed annually in 2013 and 2014 (National Avian Research Centre, unpublish report). In Israel, recent counts estimated nation-wide population size between 200 and 300 individuals (Israeli Nature Park Authority unpublished report). This non-migrant population of *C. macqueenii*, thought to be formerly widespread throughout the Arabian Peninsula, from the Levant (Harat al Hara desert from northern Saudi Arabia, Jordan, Syria, and Sinai) to Oman and Yemen, is now likely extinct across most of this range^36,37^. Yemen and Israel therefore represent the last known isolated native representatives of this non-migrant population, with potential remnants in Oman^38–40^. In other regions, and apart from reintroduced populations, breeding events are non-existent or rare, such as Jordan, where the last wild houbara nest was observed in 1963^41^.

The isolation of these ESUs at the western and southern edges of the species’ distribution range, combined with their genetic status, exacerbates their vulnerability to environmental, demographic, and genetic stochasticity. These situations warrant the need of dedicated research on these populations and the consideration of genetic rescue action such as ex situ programs and translocation^42^.

### Genetic status of migratory ESUs from eastern peripheral regions

The migratory individuals from Eastern Kazakhstan and Mongolia represent two important ESUs (ESU7 and 8, respectively) that are vulnerable to threats at the northeastern peripheral range of the species. They also differ in terms of breeding ecology and migratory patterns. According to a recent study, birds from Eastern Kazakhstan breed at higher latitudes, migrate earlier, and travel shorter distances^22^. Both ESUs are the longest migrants within the species, with individuals wintering in the southernmost range, including the Arabian Peninsula, South Iran, and Pakistan^19,22^, illustrating unique adaptive traits that are crucial to preserve. A previous study has demonstrated adaptive divergence in migratory species of falcons across the Eastern Asian range^43^, illustrating the importance of these unique environmental conditions on birds’ migratory traits. Our findings indicate that the Mongolian and Eastern Kazakhstan ESUs are genetically distinct from each other, as well as from the Greater Western and Central Asian regions (Figs. 3, 4). This translates restricted gene flow with other ESUs, reflecting demographic independence and evolutionary potential.

Recent field surveys have shown extremely low densities in Mongolia and Eastern Kazakhstan with densities lower than 0.02 houbara/km^2^ (IFHC unpublished data). The main reason for such low population size is intense hunting pressures of these birds in their wintering range^39,44^. However, genetic diversity and inbreeding levels for these two ESUs remain similar to other, less threatened ESUs, indicating that Mongolia and Eastern Kazakhstan still remain genetically stable in comparison to Yemen and Israel ESUs. The contrast between their genetic and demographic status might be due to a delay between demographic and genetic decline. This has been especially observed in other long-lived species such as the white-tailed eagle in Europe, where genetic diversity of the population persists after demographic decline because a long lifespan helps protect against genetic loss^45^. Despite facing demographic decline, *C. macqueenii*, with an average generation time of over six years and lifespans up to 12 years^18^, is likely to retain genetic diversity better than other short-lived species. The maintenance of genetic diversity might also result from connectivity events with nearby ESUs, but the observed level of differentiation suggests otherwise.

While the genetic diversity of the Eastern Kazakhstan ESU has been preserved in captivity^46^, there is an urgent need to preserve the genetic diversity of Mongolian houbara. Previous surveys have shown that *C. macqueenii* is also found in other parts of East Asia, including Inner Mongolia (China) and the Altai region (northwest Mongolia)^19^. Individuals from these locations could potentially enhance the genetic diversity of the Mongolian and Eastern Kazakhstan ESUs, thereby helping to genetically connect them. Conversely, if introgression is prevented despite colonization of individuals, it would suggest barriers to gene flow, possibly due to maladapted dispersing individuals. Given their low population densities, unique genetic and migratory patterns, and the threats faced by these ESUs, specific conservation measures are essential. This is especially true for Mongolian populations, whose genetics are not represented in conservation breeding programmes and cannot be reinforced. Genetic and demographic evaluations are needed for effective planning of their genetic rescue.

### Genetic status of migratory and non-migratory ESUs of the Greater Western and Central Asian region

The remaining ESUs (ESU3-6) from the Greater Western and Central Asian region, spanning from Iran to Central Kazakhstan, represent the core distribution range (Fig. 1). Within this region, ESU6, representing individuals following the Central migration route, stands out with the highest genetic diversity and lowest inbreeding (Fig. 2), while field surveys report higher population densities in this area^39^. The two ESUs representing individuals from Iran show significantly lower genetic diversities.

In terms of conservation, due to their differences in genetic and migratory behaviour, the ESUs representing the Central and Western migration routes must be managed separately. Similarly, the two ESUs from Iran should also be managed separately. The low genetic diversity and genetic differentiation of the Iranian population suggest some level of genetic isolation. It is believed that non-migrant houbara in southern Iran were once part of a larger population extending from the Harrat Al Ara Desert through Iraq, Iran, South Afghanistan, and Pakistan. Today, these populations are highly fragmented and likely on the brink of extinction^47^. However, little is known about their exact demographic status, as the few surveys conducted were done in winter when non-migrants are mixed with migrants on their wintering grounds. There is an urgent need for accurate demographic and genetic assessments of these remnant populations to enable effective conservation planning.

## Conclusions

Our results demonstrate that using genomics based on more representative samples of the Asian Houbara distribution range and behaviour significantly enhances our capacity to identify conservation units. This approach, combined with the AEC framework, gives better results than traditional methods for species with complex traits like *C. macqueenii*. Defining these eight ESUs is crucial for strategic planning of conservation efforts, such as genetic rescue, conservation breeding and translocations, while ensuring respect for the genetic characteristics of recipient populations to maintain local adaptations and enhance their evolutionary potential.

Future research should focus on understudied regions like Iran, Turkmenistan, Afghanistan, Pakistan, and Oman, which may provide crucial insights into the population dynamics of the species. Moreover, understanding the genetic drivers of migration, as well as the adaptive divergence across different parts of the range is essential. In this sense, transect studies are crucial for future genomic research on species like *C. macqueenii* because they offer detailed spatial and temporal insights, essential for understanding population dynamics and adaptive traits across their range. By integrating these genomic insights with conservation strategies, we can ensure the long-term survival and adaptive potential of *C. macqueenii*.

## Material and methods

### Reference genome assembly

We assembled a reference genome using genomic material obtained from a male *C. macqueenii* sampled in Yemen. This bird is one of the wild-sourced founding breeders that are part of the on-going conservation breeding programme undertaken at the National Avian Research Center (NARC) in Sweihan, Abu Dhabi (UAE), under the auspices of the Abu Dhabi government. We extracted genomic DNA from whole blood stored in EDTA at − 80 °C using NEB Monarch Genomic DNA kit (T3010) following the manufacturers ‘nucleated blood’ protocol with an elution into preheated molecular-grade water. DNA QC was performed by Qubit Fluorometer and Implen Nanospectrophotometer.

From purified DNA template, we generated high-quality, whole genome long-read sequence data using Oxford Nanopore 9.4.1 chemistry sequenced on 12 MinIon flowcells. We basecalled the reads using the super-accurate basecalling algorithm implemented in Guppy v.6.2.7 and Dorado v.7.1.4. We filtered out low-quality reads resulting in a total of 54.96 Gbp reads passing filters that we used for the assembly. We used NanoPlot v.1.4.0^48^ to quality-checked all these reads. Using NanoFilt v.2.8.0^48^, we removed all reads shorter than 500 base pairs (bp; − l 500) and/or with Q-scores below 10 (-q 10), tailcropping the remainder by 10 bp (–tailcrop 10) to remove any residual adapters present. This resulted in a total of 39.7 Gbp of trimmed data.

We generated an initial assembly using Flye v.2.9.1^49^ incorporating three rounds of Flye’s internal polishing algorithm. We performed an additional round of long-read polishing with Racon v.1.5.0^50^ and removed small haplotypic repeats with PurgeHaplotigs^51^. We evaluated assembly completeness using BUSCO v.5.4.3^52^ with the most recent avian benchmarking set (v.10) as a reference. For further assembly characterization, we assessed contig length and continuity with GFAstats v.1.3.5^53^. Finally, we used k-mer frequency distributions from ~ 20 Gb of Illumina short read sequence data to independently estimate genome size. All k-mers of 21, 26 and 31 bp were analysed using Jellyfish v.2.2.10^54^ and GenomeScope v.1.1^55^.

### Sample collection, genomic library preparation, and re-sequencing

Our samples comprised 114 individuals collected from 10 different sites across the species’ distribution range of *C. macqueenii* (Fig. 1; Tables 1 and S1). Birds were sampled as part of the conservation efforts led by IFHC. Following capture and sampling, a subset of wild birds was incorporated as founders into the conservation breeding programmes, while the remainder were released. To avoid bias and capture potential population structure linked to reproductive isolation, all birds were sampled during the breeding season, thus avoiding migrating individuals, and all locations were sampled prior to any known translocation or release of captive bred individuals. Sampling methods have previously been described^56^. Blood samples were taken from the brachial vein and stored either in 95% ethanol or on FTA cards. All sampling occurred during dedicated field expeditions conducted under agreements between IFHC and local authorities.

Blood samples were taken from the brachial vein and stored in 95% ethanol or on FTA cards. Permits to trap birds and collect blood samples were obtained from the relevant authorities in each country, under Memoranda of Understanding (MoUs) and scientific agreements. All experimental protocols were approved by the International Fund for Houbara Conservation (IFHC), a government-affiliated entity responsible for Houbara conservation and research. Sampling procedures and laboratory experiments were carried out in accordance with all applicable local, national, and international regulations and followed established ethical guidelines and best practices for avian research, ensuring the well-being of the birds throughout the process. This study is reported in accordance with the ARRIVE guidelines.

Samples were prepared as either BGISEQ or Illumina libraries at Beijing Genomics Institute (BGI) or the Oklahoma Medical Research Facility (OMRF), respectively. For the former, genomic DNA was extracted from whole blood at BGI using standard phenol chloroform protocol, while the latter was performed using spin columns following the NEB Monarch Genomic DNA Purification Kit Protocol (New England Biolabs, 2022) ensuring quality check criterias (A260/280 > 1.8, fragment size > 20 kb). At BGI, genomic DNA (1 µg) was fragmented to 200–400 bp using Covaris shearing. Size selection was performed with Agencourt AMPure XP beads, followed by end repair, adenylation, and adapter ligation. Libraries were PCR-amplified, purified, and converted to single-stranded circular DNA for nanoball (DNB) generation. DNBs containing > 300 DNA copies were arrayed on nanochips and sequenced on the BGISEQ-500 platform using combinatorial Probe-Anchor Synthesis (cPAS), generating 100 bp paired-end reads. Other libraries were prepared and short read sequence data was generated on Illumina NovaSeq (S4 flow cells) at the Oklahoma Medical Research Foundation (OMRF) yielding paired-end reads with a length of 150 bp. Samples at each facility were sequenced at 15–30× coverage; we ensured that at least one sample from each of the 10 sampling locations was sequenced at a minimum of 30× coverage.

### Bioinformatic procedure

We used FastQC v0.12.1^57^ to assess adapter contamination and the quality of short-read data. We first applied the bbsplit.sh submodule of BBmap v.35.85^58^, using a list of common Illumina adapters as a reference. We then used the bbduk.sh submodule of Bbmap with specific trimming parameters (k = 15, mink = 5, hdist = 1, hdist2 = 0, ktrim = r, qtrim = r, minlength = 36, and trimq = 14).

We mapped trimmed short-reads to our de novo reference genome using BWA mem v.0.7.17^59^. The resulting “.sam” files were converted into “.bam” files with SAMtools v.1.11^60^ and processed with Picard v.2.27.1 (Broad Institute, 2019) to clean reads, add read groups and remove duplicate reads. We obtained read depth and mapping quality metrics from final bamfiles using SAMtools and GATK v.4.2.6.1^61,62^. Individual vcf files were generated using the GATK HaplotypeCaller algorithm. As regions of excess or minimal depth frequently may contain erroneous variant calls, we calculated average depth for each individual vcf and removed all sites with coverage either less than half times or greater than twice the average coverage using VCFtools v.0.1.16^63^. These individual vcfs were then merged using CombineGVCFs, and SNPs were jointly called from the resulting gvcf using GenotypeGVCFs, both algorithms available from the GATK package. This file served as the raw set of variants for all downstream filtering and analyses.

The first round of filtering of the combined vcf was done using the call function from the BCFtools package v.1.15.1;^64^ to remove indels and non-biallelic SNPs, as well as all sites with GQ < 20. The resultant combined vcf, which consists of all high-quality SNPs found throughout the genome is called dataset SNPset#1, and was used to calculate runs of homozygosity and heterozygosity scores (see below for details).

To mitigate potential biases related to rare alleles and linked SNPs in downstream analyses (e.g., *F-stat*, DAPC, Admixture), we filtered our dataset using BCFtools^65^. SNPs with a minor allele frequency below 5% were removed, and the remaining SNPs were randomly thinned by 5 000 bp to remove linkage. The thinning distance was based on empirically determined recombination fragment sizes and linkage maps available for the collared flycatcher (*Ficedula albicollis*;^66^). Validation of this distance was conducted by comparing DAPC plots with thinning distances of 5 000 bp and 10 000 bp, showing no significant differences in terms of biological meaning. To mitigate biases from rare alleles and linked SNPs, we used BCFtools to filter out SNPs with a minor allele frequency below 5% and thinned the remaining SNPs by 5000 bp. Additionally, we addressed sex-based splitting in population structure analyses by removing SNPs associated with the Z chromosome, noting that the de novo reference assembly was created from a male, thus no reference contigs of the W chromosome were available for mapping. This was achieved by retaining the largest contigs representing N50 and excluding sex-linked contigs based on read depth differences between the sexes. This comprehensive filtering procedure resulted in the dataset called SNPset#2.

### Assessment of population genetic structure

We used the SNPset#2 for all analyses related to the population structure assessment. We first computed pairwise *Fst* between all sampling locations and generated a heatmap using the hierfstat package in R^67^. Significance testing of the *Fst* matrix was conducted using heirfstat’s boot.ppfst package with 1 000 bootstrap replicates.

We then used discriminant analysis of principal components (DAPC) to visualize individual and population differentiation, maximizing variation between samples while minimizing within-sample variation using adegenet v.2.1.8^68^. The number of principal components (PCs) was determined via cross-validation on a training dataset containing a subset of 90% of the individuals. Following Thia^68,69^, we constrained the number of informative PCs to N-1, where N is the number of locations. Each DAPC involved 10,000 cross-validation replicates for each PC retained^34^. A hierarchical iterative approach was employed to enhance population structure resolution by sequentially reanalysing the dataset after removing the most differentiated groups until no further genetic structure was observed on the first principal component axes.

Finally, we used Admixture v.1.3.0^70^ to assess population structure across the range of *C. macqueenii* and identify levels of admixture among populations. For determining the optimal K value, we ran 30 replicates from K = 2 to 9, with cross-validation (–cv) enabled for all runs, and where the maximum K is given by N-1. The most likely K was determined and visualised using the Evanno method^71^. The relative proportion of individual assignments to each cluster obtain for each level of K were visualized as bar plots in RStudio. If Admixture results showed a binary split (K = 2) due to high differentiation of a population compared to others, we removed that population and conducted a hierarchical analysis similar to the DAPC approach described earlier. Using this iterative approach combining the programme admixture and delta K, we identified K = 2 as the most likely number of clusters initially, separating Yemen from the rest. The next two iterations showed that K = 2 was also the most likely when identifying Mongolia and Eastern Kazakhstan as distinct from the rest of the sample. After removing Yemen, Mongolia, and Eastern Kazakhstan, the delta K method could not determine the most likely number of clusters, but at K = 4, we could see patterns in the relative proportion of ancestral population K that match the DAPC results.

To evaluate the influence of geographic factors on high-latitude migratory individuals (all migrants locations, excluding North Iran), following Riou et al.^28^, we analysed the relationship between longitudinal and latitudinal differences and genetic differentiation among sample locations using Spearman’s rank correlation with the pspearman package in R.

### Assessment of genetic diversity and inbreeding levels

Using SNPset#1, we used ANGSD v.0.939^72^ to derive population-level summary statistics from bamfiles, to estimate observed heterozygosity across the genome (*H*_o_) for all individuals, with results visualised using RStudio v.2022.02.3. Runs of homozygosity (ROH) indicate regions of the genome devoid of heterozygous alleles (*N*_e_ = 0), often considered to result from inbreeding. Over generations after inbreeding events, recombination during sexual reproduction will break down ROHs into shorter fragments, providing insights into the timing and intensity of inbreeding events. Both the overall percentage of the genome in ROHs and their size distribution can help differentiate between recent and ancestral inbreeding events^73^. We quantified long ROH from the SNPset#1 using a sliding window approach implemented in Plink v.1.90^74^, and using criteria described below. To account for the small size of the Asian Houbara genome (~ 1.2 GB), we used thresholds for ROH assessment that included a minimum length of 100 000 bp, at least 25 homozygous SNPs per ROH, a minimum SNP density of 1 per 100 000 bp, a sliding window comprising at least 25 SNPs and shifting one SNP at a time, allowing for three heterozygous SNPs and up to five missing SNPs per window. We restricted ROH analysis to individuals with a minimum 20 × average depth to mitigate potential adverse effect linked to SNP calling errors^75,76^.

To assess potential biases from uneven sample sizes across locations, we performed bootstrap resampling (1 000 iterations) for individual-level heterozygosity and ROH values. For each population, we randomly subsampled individuals from n = 2 up to the full sample size (e.g., n = 7 for Yemen) and recomputed mean genetic diversity (heterozygosity) and ROH estimates per iteration. This approach allowed us to evaluate the stability of our estimates across varying sampling intensities.

### Delineation of ESUs

We employed a hierarchical, multi-criteria approach to delineate ESUs by integrating genomic divergence, population structure, and ecological distinctiveness. Candidate ESUs were first identified using a pairwise *Fst* threshold of > 0.05 (p < 0.01), corresponding to restricted gene flow (Nm < 5) and indicative of demographic independence^9^. To resolve finer-scale population structure below this divergence threshold, we performed iterative ADMIXTURE (guided by Evanno’s ΔK method) and DAPC analyses, sequentially excluding the most differentiated populations to uncover hierarchical genetic patterns. ESU boundaries were defined when genomic clusters identified by both ADMIXTURE and DAPC exhibited strong concordance, indicating robust genetic structure, or when DAPC clusters aligned with patterns of biogeographic isolation and the contrasting migratory behaviours observed across the species’ range. To further validate the uniqueness of delineated ESUs, we applied the additional following criteria: intra-population diversity, inbreeding, and relatedness patterns. This conservative approach ensured that designated ESUs reflect both neutral genetic divergence and adaptive potential, as evidenced by trait divergence in ecology and migratory behaviour. To further visualise hierarchical genetic relationships among locations, we constructed a neighbour-joining dendrogram based on Euclidean distances derived from the first two principal components (PCs) of the genome-wide DAPC. This approach provides a complementary representation of genetic distances between Evolutionarily Significant Units (ESUs) without assuming a strict phylogenetic model, which is more appropriate for visualising recent population divergence within species.

## Supplementary Information

Supplementary Information.

## Data Availability

The sequencing data are accessible in the National Center for Biotechnology Information (NCBI) Sequence Read Archive (SRA) under BioProject accession number PRJNA1338100 (sample accessions SRR35788338–SRR35788451).
