# Supplementary material for: Genome wide data recover hierarchical genetic structure and help define conservation units for the threatened Asian Houbara
Source: Sci Rep. 2026 Jan 28;16:3691. doi: 10.1038/s41598-025-33691-3 (PMC12852100; doi:10.1038/s41598-025-33691-3)

Supplementary Figure S1. Overall details of the samples

| Species | Ring_num | NCBI/SRA ID | Population | Latitude | Longitude | Sampling_date | Sex | Depth | Heterozygosity | num_ROH | Total_Length_of_ROH_KB | Average_ROH_length_KB | fROH |
| --- | --- | --- | --- | --- | --- | --- | --- | --- | --- | --- | --- | --- | --- |
| Chlamydotis macqueenii | N15356 | PRJNA1346667 | C Kazakhstan | 45.14 | 68.762 | 11/06/2015 | M | 17.2487 | 0.00448 | 2 | 2373.55 | 1186.77 | 0.204437778 |
| Chlamydotis macqueenii | N15401 | PRJNA1346667 | C Kazakhstan | 44.261 | 68.337 | NA | F | 16.9419 | 0.00486 | 3 | 4846.45 | 1615.48 | 0.417432736 |
| Chlamydotis macqueenii | N15516 | PRJNA1346667 | C Kazakhstan | 44.227 | 68.387 | NA | F | 16.8634 | 0.00458 | 3 | 3778.18 | 1259.39 | 0.325420878 |
| Chlamydotis macqueenii | N15520 | PRJNA1346667 | C Kazakhstan | 44.887 | 69.161 | 17/06/2011 | F | 17.104 | 0.00464 | 1 | 1004.76 | 1004.76 | 0.086541637 |
| Chlamydotis macqueenii | N16003 | PRJNA1346667 | C Kazakhstan | 45.372 | 68.72 | 15/06/2015 | M | 17.0744 | 0.0046 | 4 | 6106.72 | 1526.68 | 0.525981871 |
| Chlamydotis macqueenii | N16037 | PRJNA1346667 | C Kazakhstan | 45.367 | 68.324 | 26/05/2015 | F | 17.1731 | 0.00491 | 2 | 2483.3 | 1241.65 | 0.213890727 |
| Chlamydotis macqueenii | N2809 | PRJNA1346667 | C Kazakhstan | 45.088 | 68.597 | 13/04/2009 | F | 17.031 | 0.00513 | 2 | 4274.64 | 2137.32 | 0.368181797 |
| Chlamydotis macqueenii | N4070 | PRJNA1346667 | C Kazakhstan | 45.252 | 68.222 | 07/06/2013 | M | 17.1618 | 0.00512 | 2 | 2199.98 | 1099.99 | 0.189487908 |
| Chlamydotis macqueenii | P2517 | PRJNA1346667 | C Kazakhstan | 45.05 | 68.543 | 02/05/2012 | M | 17.2722 | 0.00476 | 2 | 5090.63 | 2545.32 | 0.438464363 |
| Chlamydotis macqueenii | P2521 | PRJNA1346667 | C Kazakhstan | 45.013 | 65.474 | NA | M | 16.8568 | 0.00409 | 2 | 3176.64 | 1588.32 | 0.273609245 |
| Chlamydotis macqueenii | P2529 | PRJNA1346667 | C Kazakhstan | 44.976 | 68.611 | 10/04/2013 | M | 17.1577 | 0.00488 | 2 | 3345.81 | 1672.91 | 0.288180137 |
| Chlamydotis macqueenii | P2595 | PRJNA1346667 | C Kazakhstan | 42.479 | 68.093 | 15/04/2016 | M | 16.8077 | 0.00436 | 3 | 3624.96 | 1208.32 | 0.312223787 |
| Chlamydotis macqueenii | P3402 | PRJNA1346667 | C Kazakhstan | 42.673 | 67.698 | 07/04/2016 | M | 16.7642 | 0.00463 | 0 | 0 | 0 | 0 |
| Chlamydotis macqueenii | P3404 | PRJNA1346667 | C Kazakhstan | 42.829 | 67.959 | 15/04/2016 | M | 16.7659 | 0.00439 | 0 | 0 | 0 | 0 |
| Chlamydotis macqueenii | P3405 | PRJNA1346667 | C Kazakhstan | 42.384 | 67.888 | 08/04/2016 | M | 17.243 | 0.00415 | 3 | 4110.26 | 1370.09 | 0.354023477 |
| Chlamydotis macqueenii | P3406 | PRJNA1346667 | C Kazakhstan | 42.809 | 67.955 | 15/04/2016 | M | 17.2054 | 0.00442 | 3 | 3906.12 | 1302.04 | 0.336440562 |
| Chlamydotis macqueenii | P3407 | PRJNA1346667 | C Kazakhstan | 42.579 | 68.014 | 17/04/2016 | M | 17.0907 | 0.00449 | 1 | 1128.09 | 1128.09 | 0.097164253 |
| Chlamydotis macqueenii | P3408 | PRJNA1346667 | C Kazakhstan | 42.696 | 68.003 | 06/04/2016 | M | 16.3648 | 0.00433 | 0 | 0 | 0 | 0 |
| Chlamydotis macqueenii | P3409 | PRJNA1346667 | C Kazakhstan | 42.69 | 67.69 | 07/04/2016 | M | 8.1528 | 0.0046 | 0 | 0 | 0 | 0 |
| Chlamydotis macqueenii | P3421 | PRJNA1346667 | C Kazakhstan | 42.763 | 67.985 | 09/04/2016 | M | 17.0501 | 0.00475 | 2 | 2381.9 | 1190.95 | 0.205156978 |
| Chlamydotis macqueenii | P3483 | PRJNA1346667 | C Kazakhstan | 44.266 | 68.345 | NA | M | 13.3271 | 0.00446 | 1 | 1172.72 | 1172.72 | 0.101008309 |
| Chlamydotis macqueenii | ECCH00062 | PRJNA1346667 | C Uzbekistan | 39.708 | 65.163 | 16/05/2013 | M | 16.7335 | 0.00479 | 3 | 3396.78 | 1132.26 | 0.292570267 |
| Chlamydotis macqueenii | ECCH00286 | PRJNA1346667 | C Uzbekistan | 39.9 | 65.246 | 19/05/2014 | F | 16.5436 | 0.00482 | 0 | 0 | 0 | 0 |
| Chlamydotis macqueenii | ECCH00696 | PRJNA1346667 | C Uzbekistan | 39.774 | 65.31 | 05/06/2015 | M | 16.806 | 0.00517 | 2 | 2219.58 | 1109.79 | 0.191176088 |
| Chlamydotis macqueenii | ECCH01019 | PRJNA1346667 | C Uzbekistan | 39.668 | 65.195 | 22/05/2015 | F | 16.9607 | 0.00511 | 2 | 2479.85 | 1239.93 | 0.213593573 |
| Chlamydotis macqueenii | ECCH01163 | PRJNA1346667 | C Uzbekistan | 39.83 | 65.228 | 24/05/2017 | M | 16.8187 | 0.00438 | 0 | 0 | 0 | 0 |
| Chlamydotis macqueenii | ECCH01221 | PRJNA1346667 | C Uzbekistan | 39.861 | 65.202 | 22/04/2016 | F | 16.9924 | 0.00517 | 3 | 3255.89 | 1085.3 | 0.280435179 |
| Chlamydotis macqueenii | ECCH01319 | PRJNA1346667 | C Uzbekistan | 39.829 | 65.423 | 06/06/2017 | F | 17.2299 | 0.00477 | 2 | 2439.32 | 1219.66 | 0.210102657 |
| Chlamydotis macqueenii | ECCH01345 | PRJNA1346667 | C Uzbekistan | 39.746 | 65.369 | 12/06/2017 | F | 16.8738 | 0.00477 | 0 | 0 | 0 | 0 |
| Chlamydotis macqueenii | N2938 | PRJNA1346667 | C Uzbekistan | 40.793 | 65.063 | 08/04/2010 | F | 17.1322 | 0.00486 | 1 | 1135.59 | 1135.59 | 0.097810241 |
| Chlamydotis macqueenii | N2939 | PRJNA1346667 | C Uzbekistan | 41.177 | 64.823 | 23/04/2010 | F | 16.965 | 0.00491 | 0 | 0 | 0 | 0 |
| Chlamydotis macqueenii | N2941 | PRJNA1346667 | C Uzbekistan | 41.056 | 65.169 | 16/04/2010 | F | 17.0627 | 0.00489 | 5 | 12093.9 | 2418.79 | 1.041667565 |
| Chlamydotis macqueenii | N2943 | PRJNA1346667 | C Uzbekistan | 40.719 | 65.397 | 21/04/2010 | F | 17.14 | 0.00519 | 1 | 1234.26 | 1234.26 | 0.106308851 |
| Chlamydotis macqueenii | N2945 | PRJNA1346667 | C Uzbekistan | 40.839 | 65.764 | 28/04/2011 | F | 17.0244 | 0.00488 | 1 | 1181.59 | 1181.59 | 0.101772297 |
| Chlamydotis macqueenii | N2946 | PRJNA1346667 | C Uzbekistan | 40.853 | 65.794 | 11/04/2011 | F | 17.1026 | 0.00483 | 5 | 6124.21 | 1224.84 | 0.527488314 |
| Chlamydotis macqueenii | N3363 | PRJNA1346667 | C Uzbekistan | 40.709 | 65.436 | 25/04/2011 | F | 17.0631 | 0.0051 | 4 | 7345.59 | 1836.4 | 0.632687789 |
| Chlamydotis macqueenii | N3373 | PRJNA1346667 | C Uzbekistan | 39.529 | 65.756 | 25/04/2011 | F | 14.4468 | 0.00464 | 3 | 4735.72 | 1578.57 | 0.407895379 |
| Chlamydotis macqueenii | N3499 | PRJNA1346667 | C Uzbekistan | 40.853 | 65.657 | 03/05/2011 | F | 17.1404 | 0.00505 | 3 | 3470.58 | 1156.86 | 0.298926783 |
| Chlamydotis macqueenii | N12064 | PRJNA1346667 | E Kazakhstan | 47.138 | 79.63 | 03/05/2010 | F | 17.0158 | 0.00532 | 0 | 0 | 0 | 0 |
| Chlamydotis macqueenii | N12071 | PRJNA1346667 | E Kazakhstan | 47.001 | 79.877 | 17/05/2010 | M | 16.9516 | 0.00458 | 0 | 0 | 0 | 0 |
| Chlamydotis macqueenii | N12078 | PRJNA1346667 | E Kazakhstan | 47.08 | 79.841 | 30/04/2010 | F | 16.9906 | 0.00472 | 4 | 4623.03 | 1155.76 | 0.398189203 |
| Chlamydotis macqueenii | N12094 | PRJNA1346667 | E Kazakhstan | 47.144 | 80.146 | 29/04/2011 | F | 16.9807 | 0.0051 | 1 | 1167.34 | 1167.34 | 0.100544921 |
| Chlamydotis macqueenii | N12108 | PRJNA1346667 | E Kazakhstan | 47.151 | 79.86 | 04/05/2010 | F | 21.2382 | 0.00472 | 0 | 0 | 0 | 0 |
| Chlamydotis macqueenii | N12171 | PRJNA1346667 | E Kazakhstan | 46.301 | 79.064 | NA | F | 17.0051 | 0.00505 | 2 | 3929.15 | 1964.58 | 0.338424174 |
| Chlamydotis macqueenii | N2785 | PRJNA1346667 | E Kazakhstan | 47.251 | 80.041 | 29/04/2011 | F | 17.0115 | 0.00632 | 1 | 1062.21 | 1062.21 | 0.0914899 |
| Chlamydotis macqueenii | N2901 | PRJNA1346667 | E Kazakhstan | 47.102 | 79.908 | 08/05/2010 | F | 20.894 | 0.00469 | 1 | 1011.9 | 1011.9 | 0.087156617 |
| Chlamydotis macqueenii | N4373 | PRJNA1346667 | E Kazakhstan | 47.116 | 79.909 | 29/04/2011 | F | 20.7436 | 0.00462 | 1 | 1079.06 | 1079.06 | 0.092941219 |
| Chlamydotis macqueenii | P2538 | PRJNA1346667 | E Kazakhstan | 47.272 | 80.037 | 07/05/2011 | M | 16.9058 | 0.00502 | 2 | 2397.79 | 1198.9 | 0.20652561 |
| Chlamydotis macqueenii | P2541 | PRJNA1346667 | E Kazakhstan | 46.033 | 79.851 | NA | M | 17.1882 | 0.00475 | 4 | 6032.54 | 1508.14 | 0.519592625 |
| Chlamydotis macqueenii | G24810 | PRJNA1346667 | Israel | 31.219 | 34.653 | 07/13/2022 | F | 38.0255 | 0.00401 | 3 | 6076.57 | 2025.52 | 0.523385002 |
| Chlamydotis macqueenii | G24813 | PRJNA1346667 | Israel | 31.219108 | 34.652707 | 18/07/2022 | F | 36.3032 | 0.003507986 | 45 | 232134.00 | 5158.5300 | 19.9940845 |
| Chlamydotis macqueenii | G24814 | PRJNA1346667 | Israel | 31.219 | 34.653 | 07/18/2022 | F | 37.6397 | 0.00418 | 2 | 2739.34 | 1369.67 | 0.235943875 |
| Chlamydotis macqueenii | G24819 | PRJNA1346667 | Israel | 31.219 | 34.653 | 07/18/2022 | F | 39.6456 | 0.00379 | 10 | 25346.9 | 2534.69 | 2.183170325 |
| Chlamydotis macqueenii | H4347 | PRJNA1346667 | Israel | 30.811 | 34.593 | 15/02/2022 | M | 45.1218 | 0.00389 | 7 | 11381.7 | 1625.96 | 0.980324603 |
| Chlamydotis macqueenii | N4018 | PRJNA1346667 | Mongolia | 43.041 | 107.635 | 03/05/2018 | M | 17.1367 | 0.0042 | 6 | 8081.08 | 1346.85 | 0.696036756 |
| Chlamydotis macqueenii | P3744 | PRJNA1346667 | Mongolia | 43.808 | 109.233 | NA | M | 16.8449 | 0.00404 | 3 | 4760.73 | 1586.91 | 0.410049531 |
| Chlamydotis macqueenii | P3745 | PRJNA1346667 | Mongolia | 43.192 | 108.267 | 09/05/2018 | M | 21.0172 | 0.00398 | 3 | 3462.88 | 1154.29 | 0.298263569 |
| Chlamydotis macqueenii | P3746 | PRJNA1346667 | Mongolia | 42.749 | 107.574 | 04/05/2018 | M | 20.8106 | 0.00422 | 5 | 9589.88 | 1917.98 | 0.82599219 |
| Chlamydotis macqueenii | P3747 | PRJNA1346667 | Mongolia | 42.954 | 107.861 | 03/05/2018 | M | 21.1815 | 0.00419 | 2 | 2339.52 | 1169.76 | 0.201506718 |
| Chlamydotis macqueenii | P3748 | PRJNA1346667 | Mongolia | 42.722 | 107.302 | 01/05/2018 | M | 17.1767 | 0.00422 | 7 | 9275.2 | 1325.03 | 0.798888282 |
| Chlamydotis macqueenii | P3750 | PRJNA1346667 | Mongolia | 42.946 | 107.835 | 20/04/2018 | M | 16.5544 | 0.00423 | 11 | 1169.45 | 1169.45 | 0.100726658 |
| Chlamydotis macqueenii | P3752 | PRJNA1346667 | Mongolia | 43.203 | 108.297 | 09/05/2018 | M | 17.0402 | 0.00397 | 0 | 0 | 0 | 0 |
| Chlamydotis macqueenii | P3791 | PRJNA1346667 | Mongolia | 42.724 | 107.563 | NA | M | 43.6935 | 0.00383 | 3 | 4162.54 | 1387.51 | 0.358526439 |
| Chlamydotis macqueenii | P3793 | PRJNA1346667 | Mongolia | 44.878 | 97.062 | NA | M | 17.0943 | 0.00365 | 9 | 14357.8 | 1595.31 | 1.236661008 |
| Chlamydotis macqueenii | P3794 | PRJNA1346667 | Mongolia | 43.069 | 107.582 | NA | M | 34.2514 | 0.00385 | 1 | 19281.3 | 1752.84 | 1.660730188 |
| Chlamydotis macqueenii | P3798 | PRJNA1346667 | Mongolia | 42.854 | 107.648 | 27/04/2018 | M | 21.1429 | 0.00408 | 2 | 3805.71 | 1902.85 | 0.327792083 |
| Chlamydotis macqueenii | P3799 | PRJNA1346667 | Mongolia | 42.853 | 107.608 | 27/04/2018 | M | 17.1762 | 0.0042 | 2 | 2632.75 | 1316.38 | 0.226763102 |
| Chlamydotis macqueenii | P3800 | PRJNA1346667 | Mongolia | 42.962 | 107.844 | 20/04/2018 | M | 17.1267 | 0.00393 | 4 | 7383.44 | 1845.86 | 0.635947872 |
| Chlamydotis macqueenii | M03N03867 | PRJNA1346667 | N Iran | 35.799 | 54.991 | 05/05/2003 | M | 16.1151 | 0.00351 | 4 | 8362.34 | 2090.58 | 0.720262144 |
| Chlamydotis macqueenii | M03N03874 | PRJNA1346667 | N Iran | 35.733 | 54.598 | 01/05/2003 | M | 13.9417 | 0.00352 | 4 | 4408.24 | 1102.06 | 0.379688986 |
| Chlamydotis macqueenii | M03N03877 | PRJNA1346667 | N Iran | 35.809 | 55.059 | 03/05/2003 | M | 22.2535 | 0.00363 | 4 | 6379.48 | 1594.87 | 0.54947514 |
| Chlamydotis macqueenii | M03N03880 | PRJNA1346667 | N Iran | 35.715 | 54.587 | 12/05/2003 | F | 23.3457 | 0.0041 | 10 | 16271.3 | 1627.13 | 1.40147392 |
| Chlamydotis macqueenii | M03N03881 | PRJNA1346667 | N Iran | 35.822 | 55.112 | 14/05/2003 | M | 23.2148 | 0.00379 | 3 | 3723.25 | 1241.08 | 0.320689667 |
| Chlamydotis macqueenii | M03N03883 | PRJNA1346667 | N Iran | 35.812 | 55.18 | 10/05/2003 | M | 13.6112 | 0.00351 | 4 | 9332.18 | 2333.04 | 0.803796064 |
| Chlamydotis macqueenii | M03N03892 | PRJNA1346667 | N Iran | 35.675 | 54.538 | 14/05/2003 | F | 22.8138 | 0.0039 | 5 | 6834.75 | 1366.95 | 0.588688296 |
| Chlamydotis macqueenii | M03N03944 | PRJNA1346667 | N Iran | 35.679 | 54.535 | 12/05/2003 | F | 19.5228 | 0.00389 | 1 | 1023.29 | 1023.29 | 0.088137656 |
| Chlamydotis macqueenii | M12N18713 | PRJNA1346667 | S Iran | 30.1 | 54.5 | 10/04/2012 | M | 35.8628 | 0.0036 | 3 | 3353.64 | 1117.88 | 0.288854547 |
| Chlamydotis macqueenii | M12N18716 | PRJNA1346667 | S Iran | 30.1 | 54.5 | 13/04/2012 | F | 17.4032 | 0.00387 | 2 | 2795.74 | 1397.87 | 0.2408017 |
| Chlamydotis macqueenii | M12N18719 | PRJNA1346667 | S Iran | 30.1 | 54.5 | 18/04/2012 | M | 37.712 | 0.00365 | 2 | 2790.29 | 1395.15 | 0.240332282 |
| Chlamydotis macqueenii | M12N18720 | PRJNA1346667 | S Iran | 30.1 | 54.5 | 18/04/2012 | M | 21.9009 | 0.00355 | 4 | 5778.38 | 1444.59 | 0.497701405 |
| Chlamydotis macqueenii | M12N18723 | PRJNA1346667 | S Iran | 30.1 | 54.5 | 19/04/2012 | M | 30.3158 | 0.00359 | 3 | 4957.66 | 1652.55 | 0.427011437 |
| Chlamydotis macqueenii | M12N18726 | PRJNA1346667 | S Iran | 30.1 | 54.5 | 20/04/2012 | M | 25.3487 | 0.00358 | 3 | 4564.74 | 1521.58 | 0.393168589 |
| Chlamydotis macqueenii | M12N18730 | PRJNA1346667 | S Iran | 30.1 | 54.5 | 26/04/2012 | F | 22.8086 | 0.00388 | 3 | 3462.43 | 1154.14 | 0.29822481 |
| Chlamydotis macqueenii | M12N18732 | PRJNA1346667 | S Iran | 30.1 | 54.5 | 27/04/2012 | M | 25.025 | 0.00358 | 3 | 3981.56 | 1327.19 | 0.342938333 |
| Chlamydotis macqueenii | M12N18735 | PRJNA1346667 | S Iran | 30.1 | 54.5 | 30/04/2012 | M | 24.8555 | 0.00359 | 3 | 4192.7 | 1397.57 | 0.36112417 |
| Chlamydotis macqueenii | M12N18736 | PRJNA1346667 | S Iran | 30.1 | 54.5 | 01/05/2012 | F | 19.8803 | 0.00384 | 3 | 3413.61 | 1137.87 | 0.294019863 |
| Chlamydotis macqueenii | N12126 | PRJNA1346667 | W Kazakhstan | 43.122 | 52.143 | 20/04/2009 | F | 20.898 | 0.00488 | 7 | 8030.55 | 1147.22 | 0.691684524 |
| Chlamydotis macqueenii | N12133 | PRJNA1346667 | W Kazakhstan | 43.133 | 52.359 | 13/05/2009 | F | 16.9719 | 0.0053 | 4 | 4889.56 | 1222.39 | 0.421145872 |
| Chlamydotis macqueenii | N12135 | PRJNA1346667 | W Kazakhstan | 42.59 | 52.964 | 19/05/2009 | F | 20.8981 | 0.00493 | 4 | 5409.32 | 1352.33 | 0.465913658 |
| Chlamydotis macqueenii | N12137 | PRJNA1346667 | W Kazakhstan | 43.158 | 52.139 | 15/05/2010 | M | 16.7082 | 0.00457 | 0 | 0 | 0 | 0 |
| Chlamydotis macqueenii | N15623 | PRJNA1346667 | W Kazakhstan | 44.971 | 52.305 | NA | M | 29.064 | 0.00433 | 5 | 6233.3 | 1246.66 | 0.536884415 |
| Chlamydotis macqueenii | N16120 | PRJNA1346667 | W Kazakhstan | 45.074 | 52.642 | NA | M | 40.1167 | 0.00392 | 6 | 8900.8 | 1483.47 | 0.766640592 |
| Chlamydotis macqueenii | N16131 | PRJNA1346667 | W Kazakhstan | 45.039 | 52.75 | NA | M | 40.4961 | 0.00392 | 3 | 3475.06 | 1158.35 | 0.299312652 |
| Chlamydotis macqueenii | N16595 | PRJNA1346667 | W Kazakhstan | 45.022 | 52.814 | NA | M | 27.02 | 0.00449 | 0 | 0 | 0 | 0 |
| Chlamydotis macqueenii | N16692 | PRJNA1346667 | W Kazakhstan | 44.975 | 52.317 | NA | M | 44.086 | 0.00392 | 4 | 6636.54 | 1659.13 | 0.571616142 |
| Chlamydotis macqueenii | N16744 | PRJNA1346667 | W Kazakhstan | 45.049 | 52.302 | NA | M | 41.11 | 0.00387 | 8 | 14383.9 | 1797.99 | 1.238909044 |
| Chlamydotis macqueenii | P2631 | PRJNA1346667 | W Kazakhstan | 45.091 | 52.496 | NA | M | 35.0542 | 0.00632 | 1 | 1074.32 | 1074.32 | 0.092532954 |
| Chlamydotis macqueenii | P2634 | PRJNA1346667 | W Kazakhstan | 45.021 | 52.788 | NA | M | 41.8204 | 0.00388 | 4 | 6261.23 | 1565.31 | 0.539290072 |
| Chlamydotis macqueenii | P2635 | PRJNA1346667 | W Kazakhstan | 45.098 | 52.47 | NA | M | 39.1009 | 0.00392 | 3 | 3984.05 | 1328.02 | 0.343152801 |
| Chlamydotis macqueenii | P2532 | PRJNA1346667 | W Uzbekistan | 42.6 | 57.118 | NA | M | 33.6652 | 0.00401 | 3 | 3600.59 | 1200.2 | 0.310124759 |
| Chlamydotis macqueenii | P2633 | PRJNA1346667 | W Uzbekistan | 42.567 | 57.16 | NA | M | 42.1675 | 0.00392 | 6 | 8046.56 | 1341.09 | 0.693063492 |
| Chlamydotis macqueenii | P2643 | PRJNA1346667 | W Uzbekistan | 42.58 | 57.143 | NA | M | 36.9572 | 0.00406 | 4 | 6176.43 | 1544.11 | 0.531986108 |
| Chlamydotis macqueenii | P2645 | PRJNA1346667 | W Uzbekistan | 42.631 | 57.249 | NA | M | 35.9749 | 0.0041 | 0 | 0 | 0 | 0 |
| Chlamydotis macqueenii | P3300 | PRJNA1346667 | W Uzbekistan | 42.67 | 57.181 | NA | M | 45.2774 | 0.00393 | 2 | 2312.36 | 1156.18 | 0.199167383 |
| Chlamydotis macqueenii | P3412 | PRJNA1346667 | W Uzbekistan | 42.601 | 57.138 | NA | M | 52.0562 | 0.00396 | 0 | 0 | 0 | 0 |
| Chlamydotis macqueenii | P3433 | PRJNA1346667 | W Uzbekistan | 42.527 | 57.129 | NA | M | 36.3936 | 0.00391 | 0 | 0 | 0 | 0 |
| Chlamydotis macqueenii | P3499 | PRJNA1346667 | W Uzbekistan | 42.582 | 57.085 | NA | M | 30.181 | 0.00409 | 3 | 4579.21 | 1526.4 | 0.394414914 |
| Chlamydotis macqueenii | M05N06140 | PRJNA1346667 | Yemen | 16.864 | 52.021 | 27/04/2005 | M | 18.9737 | 0.00349 | 5 | 18824.7 | 3764.95 | 1.621402476 |
| Chlamydotis macqueenii | M05N06142 | PRJNA1346667 | Yemen | 16.863 | 51.958 | 25/04/2005 | M | 19.3687 | 0.00348 | 8 | 25571.3 | 3196.41 | 2.202498268 |
| Chlamydotis macqueenii | M05N06144 | PRJNA1346667 | Yemen | 16.901 | 52.081 | 24/04/2005 | M | 21.1937 | 0.0037 | 7 | 16702.9 | 2386.13 | 1.438648341 |
| Chlamydotis macqueenii | M09N11763 | PRJNA1346667 | Yemen | 16.884 | 52.118 | 12/03/2009 | F | 35.1631 | 0.00313 | 56 | 229378 | 4096.04 | 19.75670567 |
| Chlamydotis macqueenii | M09N11766 | PRJNA1346667 | Yemen | 16.847 | 51.993 | 19/03/2009 | F | 12.5922 | 0.00332 | 39 | 113769 | 2917.16 | 9.799111716 |
| Chlamydotis macqueenii | M12N15503 | PRJNA1346667 | Yemen | 16.859 | 52.017 | 03/04/2012 | F | 19.3682 | 0.00384 | 6 | 9736.92 | 1622.82 | 0.838656988 |
| Chlamydotis macqueenii | M12N15506 | PRJNA1346667 | Yemen | 16.837 | 51.991 | 24/04/2012 | F | 19.0892 | 0.00241 | 103 | 468560 | 4549.12 | 40.35784603 |


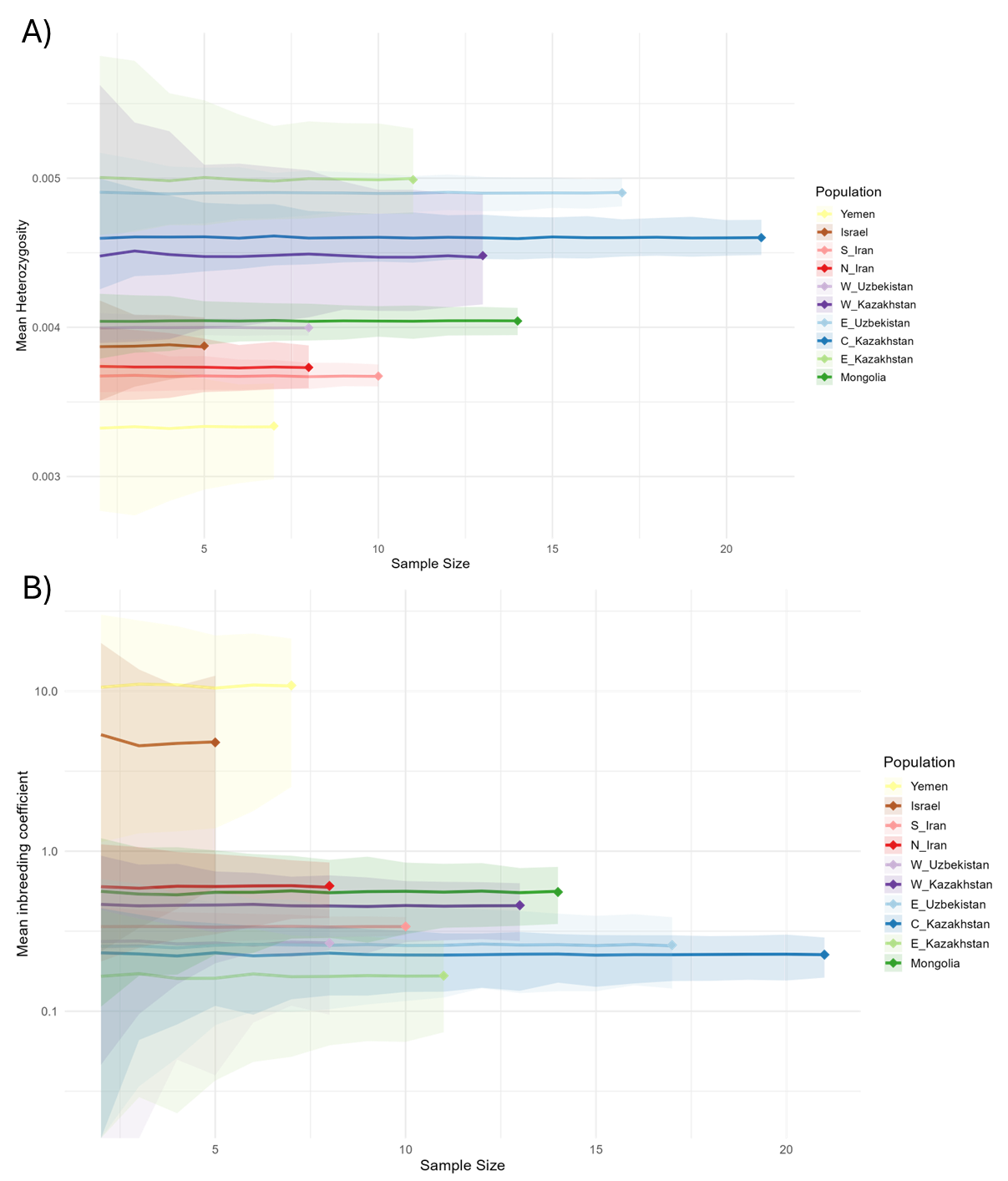


Figure S1. Bootstrap analysis of genetic diversity and inbreeding stability across houbara bustard locations. (A) Stability of observed heterozygosity (HOBS) across sample sizes. (B) Stability of inbreeding coefficients (FROH) across sample sizes. In both panels, coloured lines represent mean estimates with 95% confidence intervals (shaded regions) for distinct geographic locations. Diamonds denote final sample means. Bootstrapping (500 replicates per sample size) confirms robust mean estimates despite wider confidence intervals at small sample sizes (n < 7).

Figure S2. Admixture plots showing the genetic structure of *C. macqueenii*’s across 10 locations, with K values ranging from 2 to 9. Each vertical bar represents an individual, and the colours indicate the proportion of ancestry derived from each inferred genetic cluster K. The results illustrate the hierarchical genetic structure and the presence of distinct genetic groups within the species.


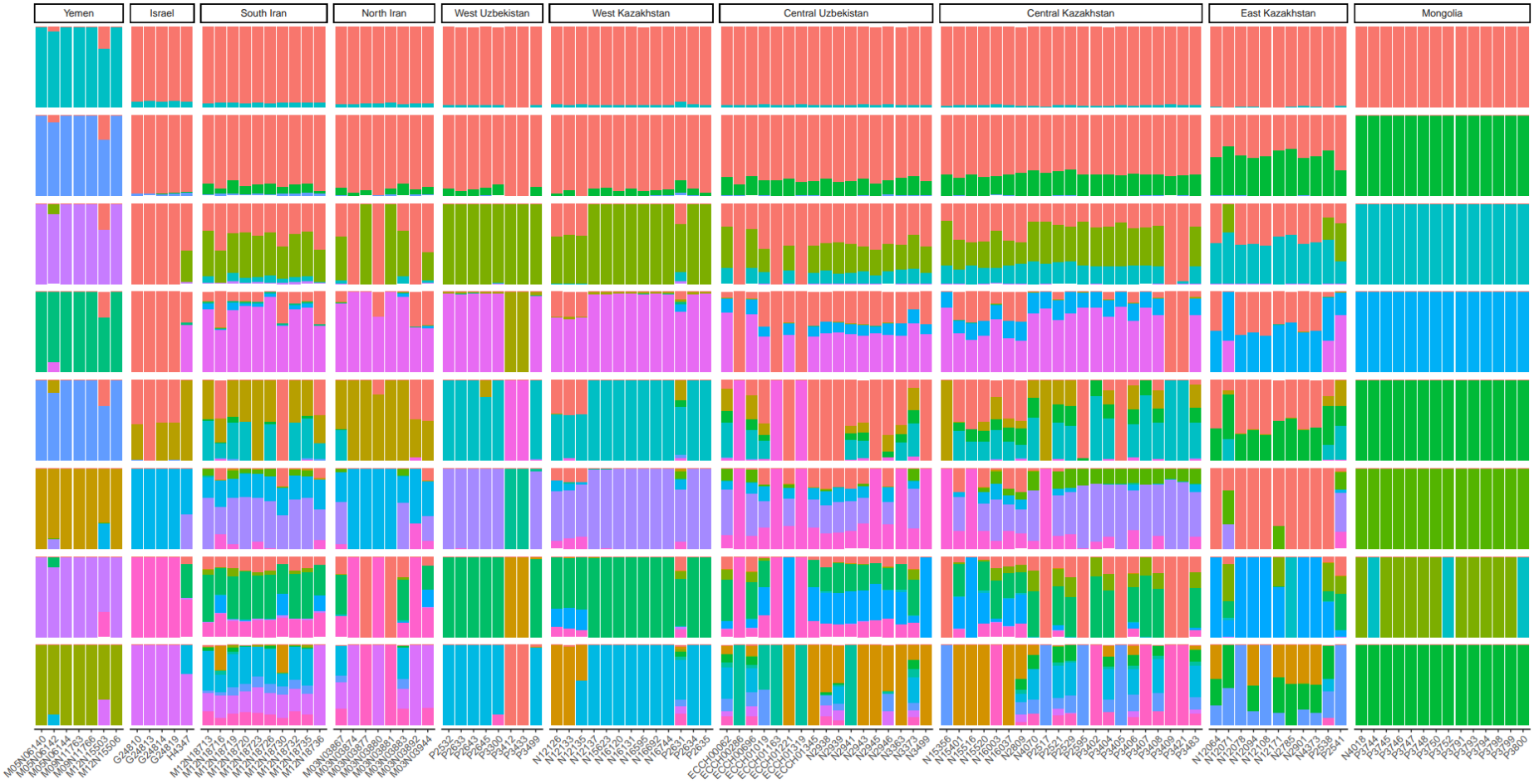

Supplement: Supplementary file 1 — Supplementary Information. [file 41598_2025_33691_MOESM1_ESM.docx]
